# Automatic liver segmentation in computed tomography using general-purpose shape modeling methods

**DOI:** 10.1186/s12938-018-0504-6

**Published:** 2018-05-29

**Authors:** Dominik Spinczyk, Agata Krasoń

**Affiliations:** Faculty of Biomedical Engineering, Silesian University of Technology, Roosevelta 40, Zabrze, Poland

**Keywords:** Liver segmentation, Single atlas based segmentation, Active Shape Model, Gaussian Process Morphable Models

## Abstract

**Background:**

Liver segmentation in computed tomography is required in many clinical applications. The segmentation methods used can be classified according to a number of criteria. One important criterion for method selection is the shape representation of the segmented organ. The aim of the work is automatic liver segmentation using general purpose shape modeling methods.

**Methods:**

As part of the research, methods based on shape information at various levels of advancement were used. The single atlas based segmentation method was used as the simplest shape-based method. This method is derived from a single atlas using the deformable free-form deformation of the control point curves. Subsequently, the classic and modified Active Shape Model (ASM) was used, using medium body shape models. As the most advanced and main method generalized statistical shape models, Gaussian Process Morphable Models was used, which are based on multi-dimensional Gaussian distributions of the shape deformation field.

**Results:**

Mutual information and sum os square distance were used as similarity measures. The poorest results were obtained for the single atlas method. For the ASM method in 10 analyzed cases for seven test images, the Dice coefficient was above 55$\%$, of which for three of them the coefficient was over 70$\%$, which placed the method in second place. The best results were obtained for the method of generalized statistical distribution of the deformation field. The DICE coefficient for this method was 88.5$\%$

**Conclusions:**

This value of 88.5 $\%$ Dice coefficient can be explained by the use of general-purpose shape modeling methods with a large variance of the shape of the modeled object—the liver and limitations on the size of our training data set, which was limited to 10 cases. The obtained results in presented fully automatic method are comparable with dedicated methods for liver segmentation. In addition, the deforamtion features of the model can be modeled mathematically by using various kernel functions, which allows to segment the liver on a comparable level using a smaller learning set.

## Background

Liver segmentation has been the subject of research by various authors for many years and is required in many clinical applications [1–3]. The most popular imaging mode is computed tomography (CT) or the contrast-enhanced CT, which are used for computer aided diagnosis (CAD) [4, 5] and planning and support of computer assisted interventions (CAI) of primary and secondary tumors in liver [6–9]. Precise liver segmentation is also crucial for selective internal radiation therapy (SIRT) [10].

The segmentation methods used can be classified according to a number of criteria. One important criterion for the division is the method of using information about the shape of the organ by a given method [11]. Simple segmentation methods that do not use shape information can be applied to different objects with a uniform intensity distribution and clearly separate the object of the interest from neighboring bodies. Such methods can be applied by selecting the values of several general parameters of the method, which can be selected on the basis of representative examples. In turn, more complex methods contain many parameters that use knowledge in the field of the shape of a segmented object.

The aim of the work is automatic liver segmentation using general purpose shape modeling methods. A new element in the work is the application of the generalized statistical model of the shape deformation method presented in [12].

## Methods

As part of the research, methods based on shape information at various levels of advancement were used. The single atlas based segmentation method was used as the simplest shape-based method. This method is derived from a single atlas using the deformable free-form deformation of the control point curves [13, 14]. Subsequently, the classic and modified Active Shape Model (ASM) was applied, using the medium body shape models and organ model [15]. The most advanced method used was generalized statistical shape models, which are based on multi-dimensional Gaussian distributions of the shape deformation field [12].

### Simple atlas method

**Fig. 1:**
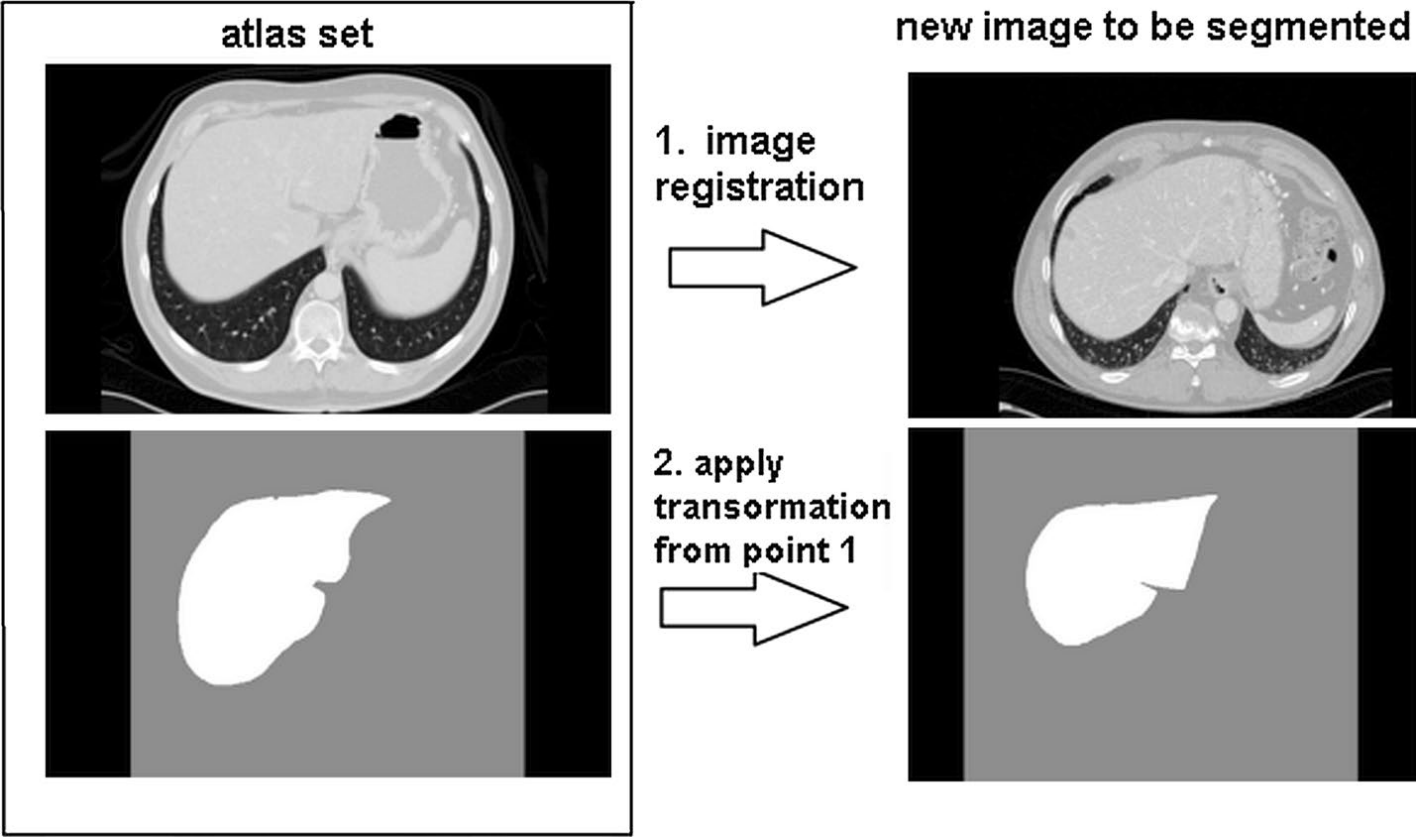
Concept behind the single atlas-based segmentation [5]

An atlas-based segmentation method propagates the segmentation of an atlas image using the image registration technique (Fig.  1).

In this paper the B-spline transformation will be used. A rectangular grid $G=K_{x} \times K_{y} \times K_{z}$ is superimposed on the image (size $N_{x} \times N_{y} \times N_{z}, K_{x}<<N_{x}, K_{y}<<N_{y}, K_{z}<<N_{z}$) which is deformed under the influence of the control points. The dense deformation is given as a summation of tensor products of univariate splines. The displacement field u(**x**) is given as:

1 $$\begin{aligned} T(\mathbf x )=\sum _{l=0}^{3}\sum _{m=0}^{3}\sum _{n=0}^{3}B_{l}(\mu _{x})B_{m}(\mu _{y})B_{n}(\mu _{z})d_{i+l,j+m,k+n} \end{aligned}$$

where $i=\lfloor x/N_{x} \rfloor -1$, $j=\lfloor y/N_{y} \rfloor -1$, $k=\lfloor z/N_{z} \rfloor -1$, $\mu _{x}=x/N_{x}- \lfloor x/N_{x} \rfloor$, $\mu _{y}=y/N_{y}- \lfloor y/N_{y} \rfloor$, $\mu _{z}=z/N_{z}- \lfloor z/N_{z} \rfloor$, $B_{l}$—represents lth basis function of the B-splne and d denotes the displacement.

Registration reffers to as a selection the set of the parameters:

2 $$\begin{aligned} \hat{\mu }= argminC(\psi ; I_F , I_M) \end{aligned}$$

where $C(\psi ; I_F , I_M)$—is the cost function related to the similarity metrics. The other important details can be found in a previous paper [5].

### Classic and modified Active Shape Model

**Fig. 2:**
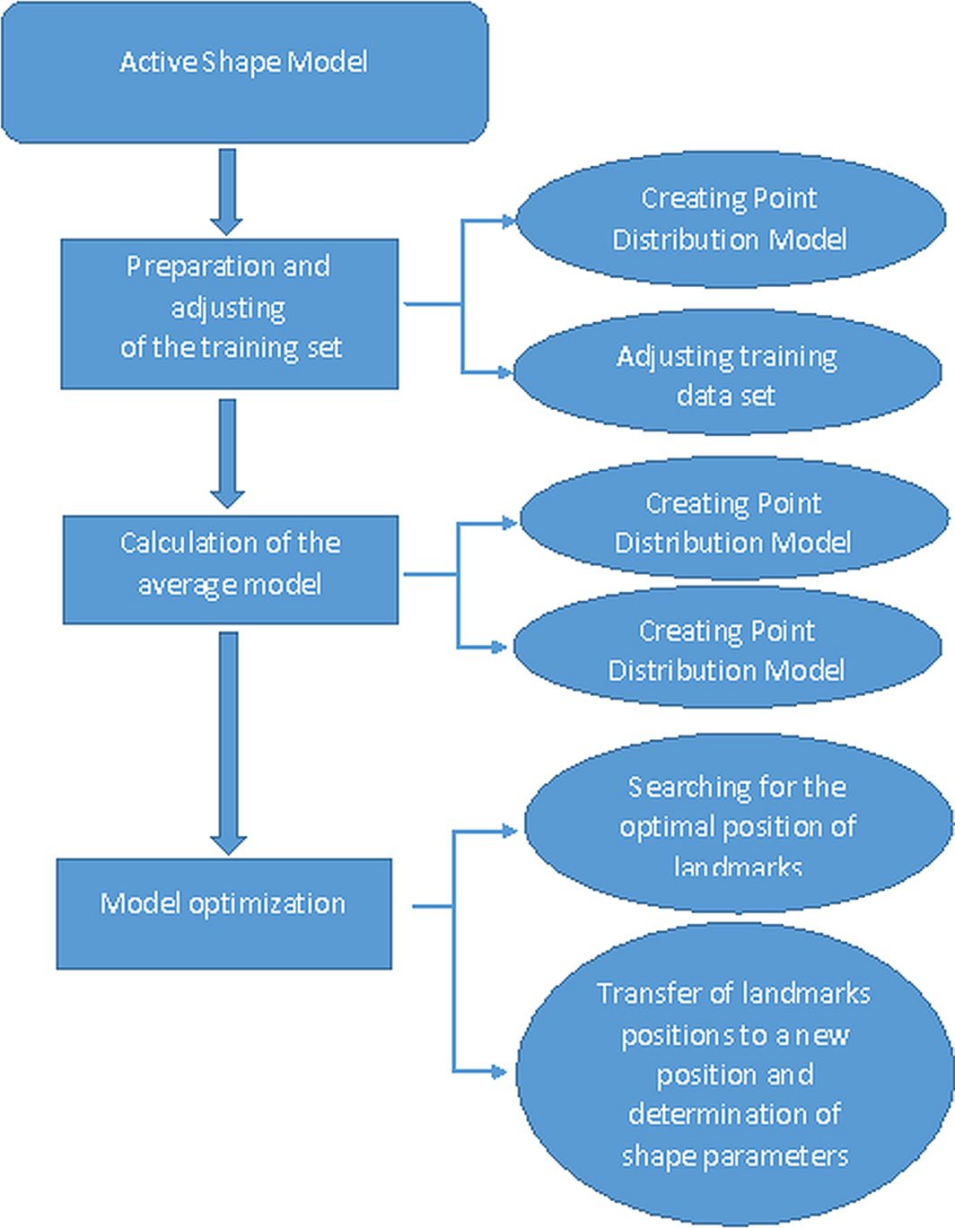
Stages of determining the shape model in the ASM method

In the 1990s, Tim Cootes and Chris Taylor introduced a new segmentation technique called the Active Shape Model (abbreviated as ASM). This method makes it possible to create a medium model of the shape of the object based on the training set, and then iteratively deform and move its boundaries so that it fits the new case as well as possible. The ASM algorithm can be divided into three main stages, which consists of: determining the training set, estimating the average (statistical) shape model, and optimizing the obtained shape model. The classic ASM method uses the point distribution model (PDM), by means of which the pattern of the segmented object is created [16]. The steps necessary to create a shape model are shown in Fig.  2

The ASM method uses the statistical analysis of coordinate positions to learn a set of landmarks. In order to be able to compare the corresponding points belonging to different shape models from the training set, user must first align the axis set. A modified Procrustes analysis is used to transform the training set data by scaling, rotation, and translation [17]. The next step in the ASM is to estimate the statistics of aligned images from the training set. First, the average shape is calculated, which can be expressed by the formula:

3 $$\begin{aligned} \overline{X}= \frac{1}{N}\sum _{i=1}^{N} X_i \end{aligned}$$

where $\overline{X}$—average shape, $X_{i}$—i-th shape, *N*—the number of alligned shape in the training set.

Ways of variability, or directions in which points of shape are going together while moving, are found using Principal Component Analysis (PCA) for deviations from the average value. For each shape from the training set, the deviations from the average $dX_i$ are estimated:

4 $$\begin{aligned} dX_i= X_i - \overline{X} \end{aligned}$$

The calculated $dx_i$ values are then used to determine the $2n * 2n$ covariance matrix *S*, where *n* is the number of landmarks

5 $$\begin{aligned} S= \frac{1}{N}\sum _{i=1}^{N} dX_idX_i^T \end{aligned}$$

The direction of the variation of the points can be described by the eigenvectors $p_i$ of the covariance matrix S. The eigenvectors of the covariance matrix corresponding to the largest eigenvalues represent the most significant variability possibilities of the values on the basis of which the matrix S was created. In addition, the ratio of the total variance to each eigenvalue is equal to the eigenvector for that value. Thanks to these properties, most of the shape changes can be translated by a small number of modes. Each shape *s* from the training set can be approximated using the average shape and the weighted sum of deviations obtained from the first *t* modes of variation:

6 $$\begin{aligned} s= \overline{X} + Pb \end{aligned}$$

where *P*—matrix of first t eigenvectors $(p_1 p_2 ... p_t)$, *b*—vector of weights for each eigenvector $(b_1 b_2 ... b_t)^T$

The use of the created average shape to the location on the images of the searched object requires that the model contains information not only about the shape, but also about the intensity levels and texture of the objects. For this purpose, the models of the appearance of the intensity level (Gray-Level Appearance Model) are determined [18]. When creating appearance models, statistical values of gray levels are analyzed in the areas around each landmark determined in previous stages of the ASM algorithm. By assuming the ASM method, the orientation points correspond to specific locations on objects in all learning images, which is why the designated gray level patterns in different images around landmarks should be similar. The adjustment of the previously estimated average shape to the new case can be broadly divided into two steps repeated in an iterative manner:

- Creating a series of hypotheses giving approximate locations of model points,
- Refining each hypothesis and choosing the best one.

The Active Shape Model method has undergone many different modifications and improvements. The main problem of the ASM method is generating learning data that meets the following conditions:

- In every set of points there must be n corresponding landmarks,
- Each landmark must lie in the image on the edge of the object the model is created,
- Each set of n points must form a spatial grid consisting of polygons (creating a vertices-faces representation).

In this paper the ASM with the optimal features is used. This is an alternative to the classic ASM abandoning the creation of the Gray-Level Appearance Model using standardized derivative profiles and without estimating the cost function based on the Mahalanobis distance in favor of the algorithm which allows you to move the landmarks towards a better location during optimization along a perpendicular direction to the object edge [15]. The authors assumed that the best location is one for which everything on one side of the profile is outside the object, and everything on the other side is in the middle of the object. In order to determine whether a given location is in or outside the object, the probability is determined for the area around each landmark, on the basis of which the classification is made. The decision on the best new location of the point is made by the non-linear classifier of the k-nearest neighbors classifier (kNN-classifier).

The authors of the article [19] proposed using the iterative closest point method (ICP) to generate a training set. Behiels et al. [20] proposed to improve the way the shape of the model is adjusted to the new case in ASM. In classic ASM, the position and shape parameters are corrected at each iteration by minimizing the sum of the squares of differences between model points and points estimated in a given iteration. This criterion of matching with the smallest squares is sensitive to outliers, which results in poorer results. When the outlying points attract further points towards it, the location of the landmarks can longer be improved in the next iteration. For this reason, it has been proposed to correct the suggested *dX* displacements before updating the position and shape parameters.

In order to prepare the data, all the images were subjected to the initial phase of data processing resulting in the generation of volumes on which the liver is visible in 60 cross-sections. The initial processing phase can be divided into two stages. In the process of creating models of liver shape, prepared, segmented images were used. Based on these images, the edge of the liver was marked. Then 20–100 points lying on the designated border were selected. For the first four and last liver cross-sections, the number of landmarks changed by 20 points, while for others the number was constant and amounted to 100. Three image resolution coefficients were used in the calculations: 0.25, 0.5 and 1. For each of them, five iterations with unchanged parameters were made. During results generation, the length of the profiles was 8 pixels, the number of determined landmark positions was 6 and the limits of the own values to 3.

#### Generalized statistical shape model

The statistical generalized model—Gaussian Process Morphable Model (GPMM) based on the decomposition of the main components is a linear model that can be presented in a parametric manner [12]. The shape of the object can be represented as a folding of the deformation of an object with a reference shape:

7 $$\begin{aligned} s= {X + u(X)|X \in \Gamma _R} \end{aligned}$$

where *s*—the shape of the object (the vector of the coordinates of points lying on the surface of the model), *X*—shape vector, *u*—$\Omega \rightarrow R^3$ deformation field (vector field of the shape difference), $\Gamma _R$— reference shape.

The deformation field is modeled as a multi-dimensional Gaussian process:

8 $$\begin{aligned} u = GP(\mu ,k) \end{aligned}$$

where *GP*—Gaussian process, $\mu$—$\Omega \rightarrow R^3$ average deformation, *k*—$\Omega \times \Omega \rightarrow R^{3 \times 3}$ covariance function or kernel function.

The idea of a statistical model of a shape is based on the decomposition of the principal components and consists in the representation of a multi-dimensional Gaussian process in the form of a low-dimensional sum of *r* of the leading base functions:

9 $$\begin{aligned} u = \mu + \sum _{i=1}^{r} \alpha _i \sqrt{\lambda _i}\phi _i \end{aligned}$$

where $\phi _i$—i-th basic function, $\lambda _i$—variance of shape corresponding to the i-th base function $\phi _i$

**Fig. 3:**
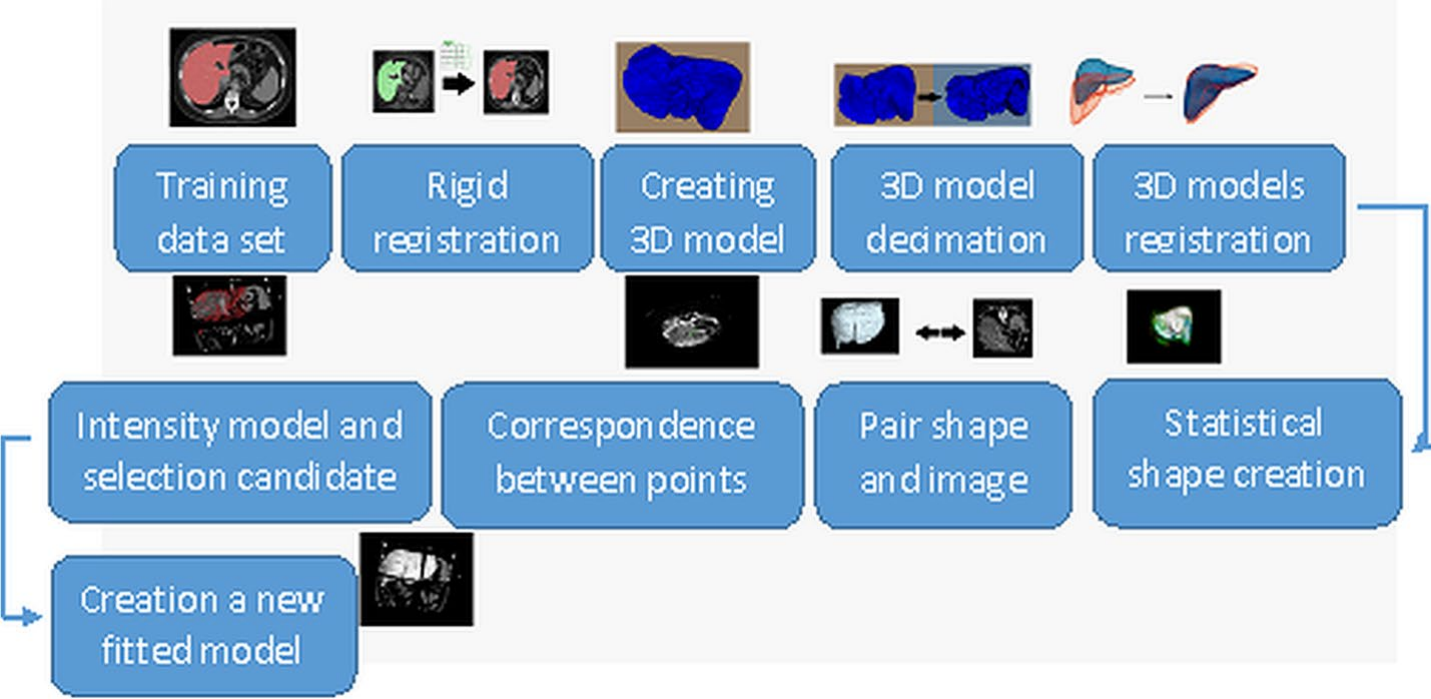
The steps for liver segmentation using generalized statistical shape model

If the modeled shape of the object is characterized by high smoothness, then only a few basic functions are needed to create a good approximation of the shape - hence the name of the low-dimensional representation of the shape. The challenge with this approach is to effectively calculate the value in the sense of computational complexity, therefore, with a larger number of model points, Nyström approximations are used [21]. One of the major advantages of a generalized statistical model, based on a multidimensional Gaussian process, in relation to the classical statistical model is the freedom to select the function of the nucleus. For a small training set, shape statistics can be represented by modeling properties of the kernel functions known from the image registration process such as radial functions or spline functions. The method presented above was adapted for the needs of segmentation of the liver organ - using the following main stages:

- Rigid registration of input images
- Finding correspondence in example sample vectors
- Building a statistical shape model based on sample shapes
- Segmenting of a new case

whose main elements have been characterized below. The more detailed steps are presented in Fig.  3.

#### Rigid registration of input images

The data collection of abdominal CT reference images together with the expert outlines of the liver organ is subjected to a rigid registration of the similarity based on the minimization of MI, which brings the entire set to one common coordinate system. This is a necessary step because the generalized statistical model simulates shape deformations.

#### Finding correspondence in example shape vectors

Ensuring the adequacy of points in shape vectors is a prerequisite for using input data in the further stages of building a Gaussian process. Finding the appropriateness of points in a set of shapes from a training set proceeds in the following main steps:

1. Selecting of one of the shapes as a reference shape.
2. Building a Gaussian process on a reference model using the kernel function for a smooth deformation model.
3. Distributing the Gaussian process to *r* Karhunen-Loe ve coefficients.
4. Registering of the model to individual shapes from the training set using the model development as a function of regularization in the registration task consisting of minimizing the distance measure.

#### Building a statistical shape model based on sample shapes

The use of the selected kernel functions for the Gaussian process modeling shape deformation does not ensure that knowledge about the subject shapes of the modeled object is taken into account. The perfect model of shape should only be deformed to shapes that can occur in reality, which is the main assumption behind the construction of statistical shape models. In the presented approach, the model is taught this property based on a set of sample shapes $\Gamma _1, ..., \Gamma _N$. After providing correspondence between the reference shape $\Gamma _R$ and all the sample shapes, we obtain a set of deformation fields:

10 $$\begin{aligned} \{u_1,...,u_N\},u_i: \Omega \rightarrow R^d \end{aligned}$$

where $u_i(X)$—the deformation field that maps the point of the reference surface X $\Gamma _R$ to its corresponding point $u_i(X)$ on the $i-th$ surface.

The Gaussian process $GP(\mu _{SM},k_{SM})$, which models this characteristic deformation, is obtained by the average estimation of its parameters:

11 $$\begin{aligned} \mu _{SM}(X) = \frac{1}{N}\sum _{i=1}^{N} u_i(X) \end{aligned}$$

and

12 $$\begin{aligned} k_{SM}(X,Y) = \frac{1}{N-1}\sum _{i=1}^{N}( u_i(X)-\mu _{SM}(X))( u_i(Y)-\mu _{SM}(Y))^T \end{aligned}$$

#### Segmentation of a new case

After constructing a generalized model of probabilistic distribution of shape deformation based on shapes from the training set, such a model can be used to segment the object in a new input image. In this case, the properties of a continuous multidimensional Gaussian process. The algorithm adjusting the model minimizes the Mahalanobis distance between the given and the average profile along the normal direction to the model. The main difference between ASM and GPMM is the generation of candidates for the new location of the points matching the image of the model. In the classic ASM approach, candidates are found along the normal vector to a model with a given length parameter. In the case under consideration, candidates are generated as samples from the Gaussian distribution of liver shape deformation built on the training set. Therefore, the new position of the model point does not have to be on the normal vector, but results from the probability distribution of the shape deformation field. In order to preserve the possibility of comparing the results in the considered scenarios, the same number of model points was used as in the case of the classical ASM algorithm.

#### Input data set

For the liver segmentation, 20 CT images of the abdominal cavity with a resolution of 512 by 512 pixels available in the SILVER07 database were used [22]. The number of cross-sections on the images ranged from 64 to 394, while their thickness ranged from 0.7 to 3 cm. Each volume also contained a binary image with a segmented liver organ made with the help of radiologists (experts).

#### Quantifying segmentation quality

Ground truth, which are usually manually segmented by a human expert, is necessary to quantify segmentation accuracy.

As similarity index the following measures are used:

- Dice similarity coefficient (DICE) defined as:
  13 $$\begin{aligned} DICE(I_{F-Seg},I_{M-Seg}(T))= 2\frac{|I_{F-Seg} \cap I_{M-Seg}(T)|}{|I_{F-Seg}|+|I_{M-Seg}(T)|}*100\% \end{aligned}$$
  where $I_{F-Seg},I_{M-Seg}$ present two segmentations and |$\cdot$| denominates the number of voxels inside the segmentation,
- Mean surface distance (MSD) is defined as:
  14 $$\begin{aligned} MSD(I_{F-Seg},I_{M-Seg}(T))= \frac{1}{n_{X}+n_{Y}}\sum _{i=1}^{n_X} d_i\sum _{j=1}^{n_y} d_j \end{aligned}$$
  where $n_{X},n_{Y}$ represent the number of voxels on the two segmentation surfaces respectively, and *d* are the closest distances from each voxel on the surface to the other surface. The value of this measure helps to identify distance between registered surfaces.
- Hausdorff distance is defined as:
  15 $$\begin{aligned} H(I_{F-Seg},I_{M-Seg}(T))= \max \left\{ \sup _{x\in N_X}\inf _{y\in N_Y}d(x,y),\sup _{y\in N_Y}\inf _{x\in N_X}d(x,y)\right\} \end{aligned}$$
  where $N_{X},N_{Y}$ represent the voxels on the two segmentation surfaces respectively, and $d_i$ and $d_j$ are the closest distances from each voxel on the surface to the other surface.

## Results

**Fig. 4:**
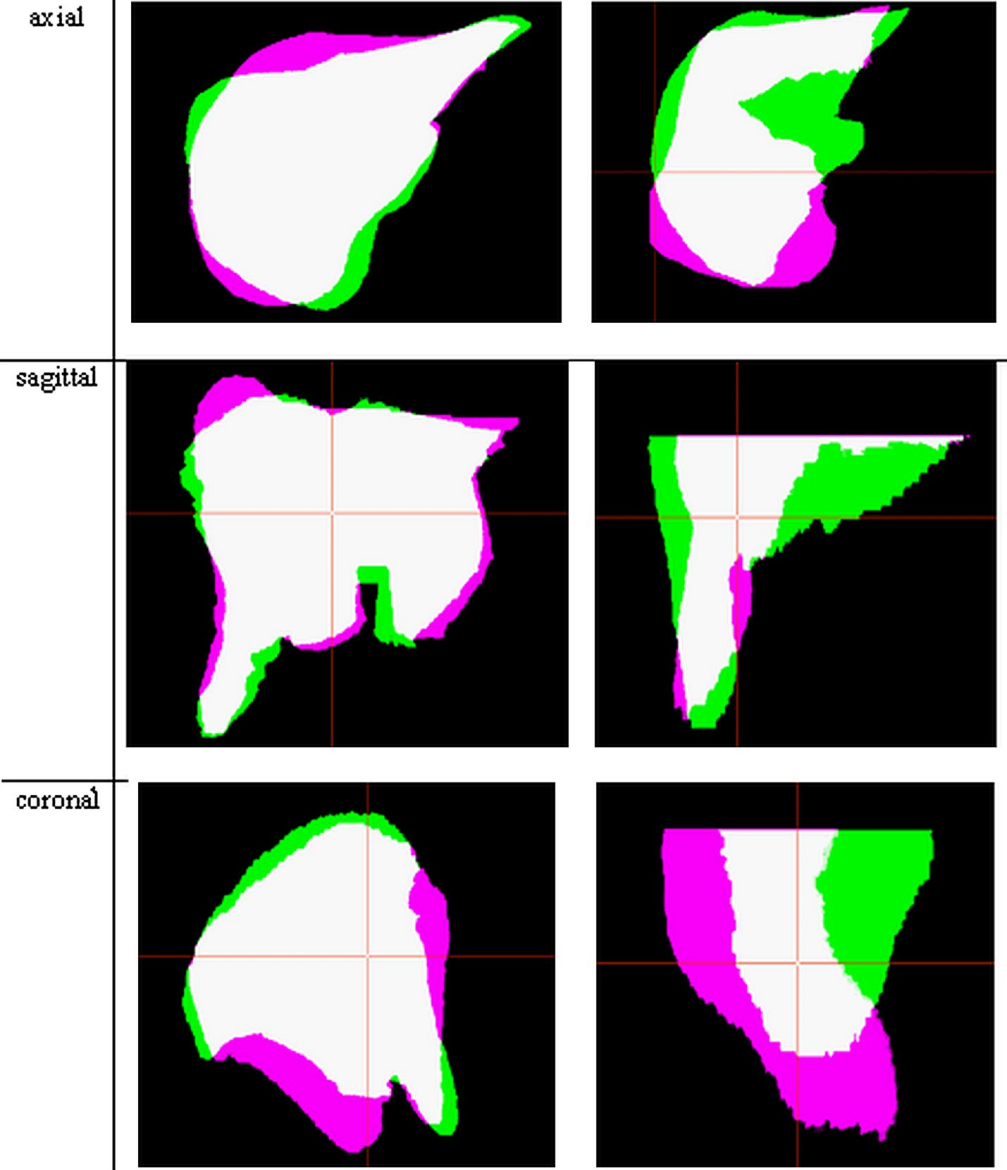
The best (left) and worst (right) result of liver segmentation according to the criterion of the dice coefficient: expert delineation (pink color), automated results (green color), common part (white color)

The segmentation quality measures defined above (DICE, MSD, Hausdorff) were used to present the results. Table 1 presents a quantitative summary of the obtained results. Statistical analysis of the results of the DICE coverage coefficient was performed. After demonstrating the lack of normal distribution (Shapiro-Wilk test), the Wilcoxon test was used to verify the median equality. Statistically significant differences in median DICE coefficient for the method of generalized statistical form factor in relation to Active Shape Model based on medium shape and a single atlas were demonstrated. The graphical representation of the results below presents the best and worst result of liver organ segmentation (according to the criterion of the DICE—Fig. 4).

**Fig. 5:**
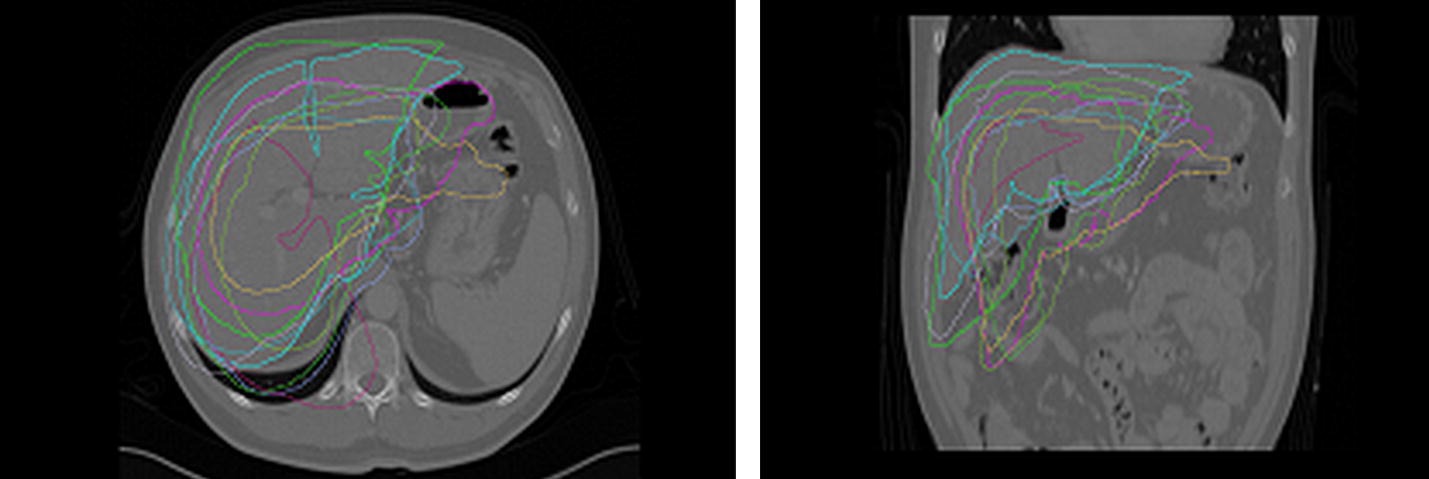
Drawings of registered liver surfaces in projections: axial (left) and coronal (right) using the Rigid registration method for training data set

Figure 5 presents drawings of registered liver surfaces in axial and coronal projections using the rigid registration method for training data set.

**Table 1:** Quantifying segmentation results according using methods - shape model points was limited to 10,000 and to 10 cases in training set

Similarity measure	Single atlas SSD	Single atlas MI	Classical ASM	Modified ASM	GPMM 10,000 pts	GPMM 19,000 pts	GPMM 40,000 pts
MSD (mm)	3.49	3.08	2.91	2.85	2.79	1.95	1.7
H (mm)	6.02	4.65	574.29	572.95	4.32	3.7	2.9
DICE (%)	0.49	0.59	0.57	0.57	0.74	0.85	0.885

## Discussion

Liver segmentation using the methods used (single-atlas method, ASM and generalized statistical model of shape deformation) showed differences in results depending on the approach used (Table 1). The more complex the shape model used, the better the results. The poorest results were obtained for the single atlas method, with the measure of MI from the SSD measure being a better measure of similarity. In the case of the ASM method, in 10 analyzed new cases for seven test images, the DICE coefficient was obtained above 55$\%$, of which for three of them the coefficient was over 70$\%$, which placed the method in second place.The best results were obtained for the method of generalized statistical distribution of the deformation field. The best results had a DICE of 75$\%$. Various specialized methods of liver segmentation are described in the literature [2, 23, 24]. They present DICE coefficients between 85 and 95 $\%$. The lower value of DICE in the presented approach can be explained by the use of general-purpose shape modeling methods, with a large variance of the shape of the modeled object—the liver. Due to the database of contoured expert cases, the training set was limited to 10 cases and number of shape model points was limited to 10,000.

To confirm the competitiveness of the proposed method, the approach used was compared with the three best methods dedicated to liver segmentation indicated in the MICCAI challenge [22]. In these studies we demonstrated the scalability of the proposed method. We increased the number of model points holding four times with an average DICE rating of 88.5$\%$ and scoring according to the MICCAI Challenge criterion 65 pts. The authors of these articles shared the results they obtained. The best methods [25–27] scored 73, 59 and 43 pts respectively. Compared with the dedicated methods for liver segmentation with MICCAI challenge [22], the method took the 2nd place. The first place was taken by a method containing both a deformation based on Free-Form Segmentation statistics and shape statistics. However, this method required advanced assumptions and calculations related to Constrained Free-Form Deformation for a typical intensity distribution around the liver. In addition, the model the statistical was built on a four times larger training set [25]. The presented method does not contain any stages characteristic for the liver and is also fully automatic. In addition, the deforamtion features of the model can be modeled mathematically by using various kernel functions [12], which allows to segment the liver on a comparable level using a smaller learning set.

In the future, it is planned to create a deformation model on a larger training set.

## Conclusions

The aim of the work is automatic liver segmentation using general purpose shape modeling methods. A new element in the work is the application of the generalized statistical model of the shape deformation method presented in [12]. Due to the clinical relevance of the problem of automatic liver segmentation and the existence of many highly specialized methods and the development of general-purpose methods of statistical shape modeling, research on the effective use of general-purpose methods to support clinical goals seems to be one of the many significant directions of development of modern biomedical engineering.

In the presented approach, a DICE of 88.5$\%$ potentially indicates an the possibility of effective use of the modern general-purpose methods while building the most representative dictionaries that cover the variability of anatomical shapes in the patient population. The obtained results in presented fully automatic method are comparable with dedicated methods for liver segmentation. In addition, the deforamtion features of the model can be modeled mathematically by using various kernel functions, which allows to segment the liver on a comparable level using a smaller learning set.
